# Long-term effectiveness of local BM-MSCs for skeletal muscle regeneration: a proof of concept obtained on a pig model of severe radiation burn

**DOI:** 10.1186/s13287-018-1051-6

**Published:** 2018-11-08

**Authors:** Christine Linard, Michel Brachet, Bruno L’homme, Carine Strup-Perrot, Elodie Busson, Michel Bonneau, Jean-Jacques Lataillade, Eric Bey, Marc Benderitter

**Affiliations:** 1 Institute of Radiological Protection and Nuclear Safety, B.P. n°17, F-92262 Fontenay-aux-Roses, France; 2 Department of Plastic Surgery, Military Hospital of Percy, Clamart, France; 3 Unité des Médicaments de Thérapie Innovante, Centre de Transfusion Sanguine des Armées, Clamart, France; 4 Centre of Research in Interventional Imaging, National Institut of Agronomic Research, Jouy-en-Josas, France; 5 Unité de Thérapie Tissulaire et Traumatologie de Guerre, Institut de Recherche Biomédicale des Armées, Clamart, France

**Keywords:** BM-MSC, Muscle, Pig, Irradiation, Regeneration

## Abstract

**Background:**

Medical management of the severe musculocutaneous radiation syndrome involves surgical intervention with debridement of necrotic tissue. Even when skin excision is replaced by specific plastic surgery, treatment of the muscle radiation injury nonetheless remains difficult, for it involves a massive muscle defect in an unpredictable environment, subject to inflammatory waves weeks to months after irradiation, which delay healing and predispose the patient to the development of fibrous scar tissue. In this study, we investigated the long-term effect of local injections of bone marrow-derived mesenchymal stromal cells (BM-MSCs), combined with plastic surgery, to treat muscle necrosis in a large animal model.

**Methods:**

Three months after irradiation to the rump, minipigs were treated by excision of necrotic muscle tissue, vascularized flap surgery, and four injections with or without local autologous BM-MSCs, performed weekly. The quality of the muscle wound healing was examined 1 year post-surgery.

**Results:**

The skeletal muscle surgery without MSC treatment led to permanent deposition of collagen 1 and 3, decreased myofiber diameter, failed muscle fiber regeneration, a reduced number of capillaries, and the accumulation of high calcium and fat. In animals treated by surgery and MSC injections, these indicators were substantially better and demonstrated established regeneration. MSC therapy acts at several levels by stimulating growth factors such as VEGF, which is involved in angiogenesis and satellite cell pool maintenance, and creating a macrophage M1/M2 balance.

**Conclusion:**

Thus, cell therapy using BM-MSCs is an effective and safe way to improve recovery of irradiation-induced skeletal muscle damage without signs of long-term degeneration.

## Background

The regeneration of skeletal muscle is a natural process after minor trauma, for this muscle has a remarkable capacity for regeneration [1] due to the presence of stem cells. Specifically, satellite cells lie quiescent under the basal lamina of muscle fiber until activated in response to injury, when they leave their niche and proliferate before differentiating into myoblasts and fusing into new myofibers [2]. After severe musculocutaneous irradiation exposure (absorbed dose greater than 25 Gy, for which necrosis is inevitable), lesions may therefore quickly extend beyond the cutaneous plane alone, involving the underlying tissues, particularly the muscle [3]. In this case, natural muscle regeneration is highly compromised. Skeletal muscle regeneration is a complex process characterized by inflammation, extracellular matrix (ECM) remodeling, angiogenesis, and myofiber growth [1, 4, 5]. Impairment of any one of these processes can lead to incomplete skeletal muscle regeneration, which in turn leads to fatty degeneration [5]. Particularly after injury, the amount of ECM, composed primarily of collagen 1 and 3 [6], may increase dramatically relative to muscle fibers and form fibrotic scar tissue compromising myofiber contractility and tissue architecture [7]. Accordingly, muscle fibrosis presents a challenge to clinicians because it impairs complete muscle recovery, compromises both muscle function and structural integrity, and increases the likelihood of reinjury [8]. The functional properties of skeletal muscles depend on the maintenance of a complex framework of myofibers, motor neurons, blood vessels, and the extracellular connective tissue matrix [1].

After therapeutic or accidental overexposure to ionizing radiation, the treatment of musculocutaneous radionecrosis is complex. It requires surgical excision, with debridement of the necrotic tissue (skin and muscle), followed by different approaches including skin graft, by dermal substitute grafts, and more recently by flap rotation [3]. The difficulty of this surgery is that it involves a massive muscle defect lying in an unpredictable environment in which inflammatory waves can come weeks to months after injury, thus delaying healing and predisposing the subject to the development of fibrous scar tissue. In addition, regeneration is also limited in situations of irreversible muscular atrophy following long-term peripheral nerve injury [9]. In recent years, researchers have paid some attention to therapeutic strategies that can improve skeletal muscle healing and regeneration through the transplantation of muscle stem cell-derived myoblasts or myogenic cells in models of muscle injury [10]. But their use has been limited by the difficulty of obtaining the necessary number of these cells for effective transplantation due to the cultivation time required to generate muscle stem cells.

Mesenchymal stem cells (MSCs) have become one of the most exciting ways for tissue regeneration due to their high plasticity and their capacity for proliferation and multilineage differentiation. They are recognized as a valuable source of cells to enhance muscle regeneration. Their regenerative capacity has been validated in several animal models of muscular dystrophy and trauma [11]. More recently, intramuscular autologous injection of adipose-derived stem/stromal cells (ASC) was shown to be effective in preventing muscle inflammation and decreasing the area of irradiation-induced fibro-necrosis at 11 weeks post-treatment, thus enabling regeneration [12].

The unpredictable spatiotemporal course of the substantial inflammatory waves due to irradiation raises two primary questions: regeneration and long-term maintenance of healing, either without inflammation and/or with renewed fibrosis. We recently showed in a preclinical model that irradiated skin could be successfully and lastingly remodeled after excision of necrotic cutaneous tissue by vascularized flap surgery combined with BM-MSC treatment [13]. In this study, we investigated the long-term effects of post-irradiation treatment by bone marrow-derived MSCs on muscle regenerative capacity, muscle fibrosis, and angiogenesis in a large preclinical model after surgical excision and debridement of necrotic tissue (skin and muscle).

## Methods

### Animal care

FBM minipigs, 12 months old and weighing about 20–25 kg (from La Ferme du Noyer, Bretoncelles, France), were placed in individual pens (21 °C, 12-h/12-h light-dark schedule) in which they received solid food and had access to water ad libitum.

### Irradiation

Anesthetized (1.5% isoflurane in oxygen) animals received a total X-ray dose (Photon 4 MV, Linear Accelerator Alphee, IRSN, France) of 90 Gy, delivered in one external beam to a 5 × 5 cm^2^ area of the rump. The beam field was aligned with the area to be irradiated by onboard lasers. Physical dosimetry was evaluated by thermoluminescent dosimeters, with alumina powder placed in the irradiated area.

### Surgery and cell therapy

**Fig. 1:**
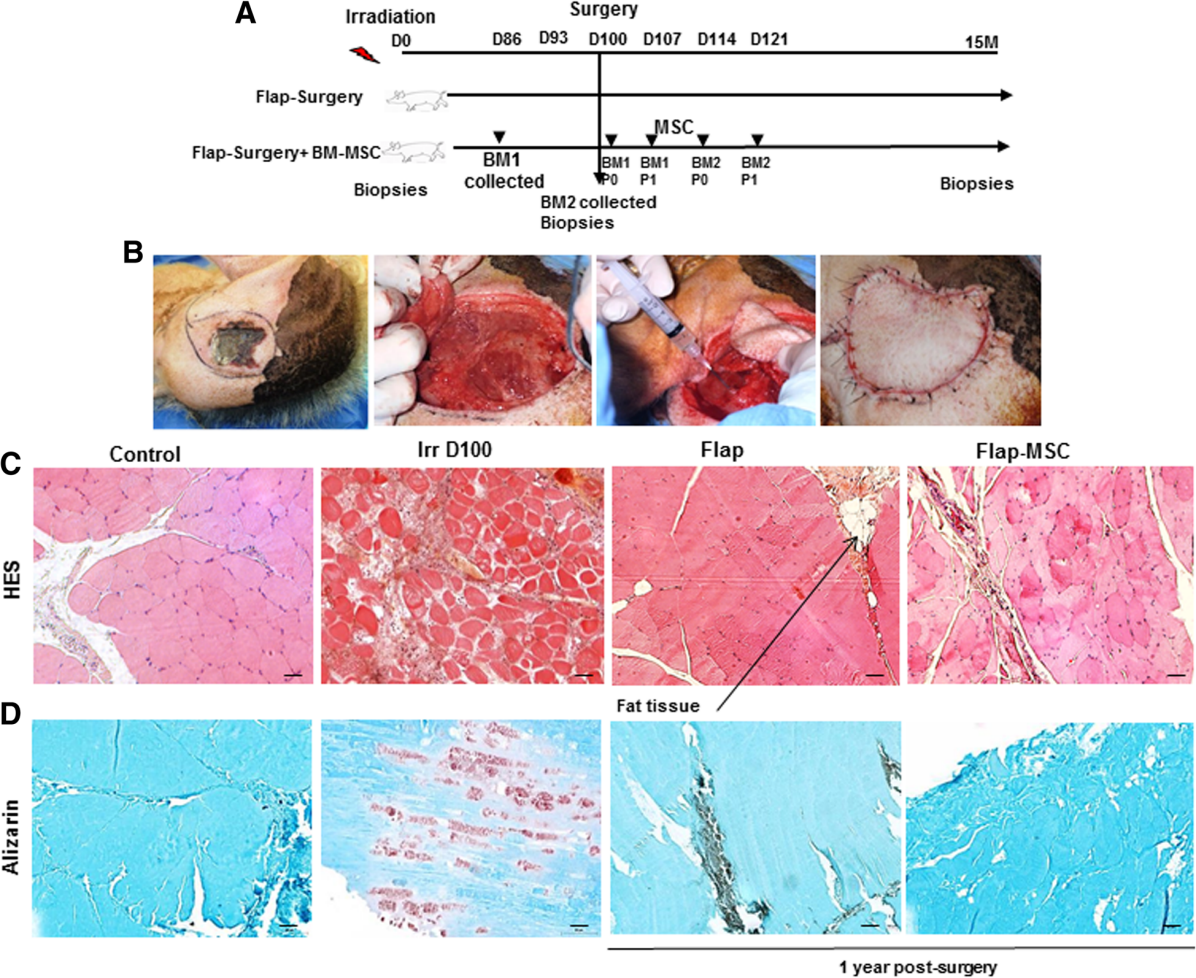
Development of a pig model of musculocutaneous radiation lesion. **a** Experimental design. **b** Photographs at surgery for fibrosis/necrosis. The skin and muscular necrosis and underlying tissues up to healthy muscle were excised, and all deep fibrosis was removed until bleeding and muscle contraction occurred. A pedicled fasciocutaneous perforator flap was elevated and closed at the site with absorbable suture. **c** Histological characteristics of the skeletal muscle after hematoxylin and eosin staining. **d** Alizarin-stained transverse sections of nonirradiated skeletal muscle (control group), irradiated skeletal muscle on the day of surgery (Irr D100 group), and 12 months after flap surgery with (flap-MSC group) and without (flap group) MSC treatment. The flap group showed large areas of fat cell infiltration, which is absent in the groups with MSC treatment. Scale bar 100 μm

This study concerned the same animals previously described [13] and included eight pigs divided into two groups: four irradiated pigs received a fasciocutaneous perforator flap (flap-only group), while four other irradiated pigs received this flap as well as repeated local administrations of autologous bone marrow-derived MSCs (flap-MSC group) (Fig. 1a). Both groups first underwent excision of skin necrosis and underlying tissues up to healthy muscle 100 to 110 days after irradiation, with all deep fibrosis removed until bleeding and muscle contraction occurred (Fig. 1b). In both flap groups, a magnifying lens was used to deepen the incision down into the muscle fascia and allows us to identify one or more musculo- or septocutaneous perforators. A skin paddle was then designed, and a pedicled fasciocutaneous perforator flap elevated. The elasticity of the surrounding skin allowed direct closure of the donor site with absorbable suture. In the flap-MSC group, the same surgical flap technique was supplemented with injections (four, each 1 week apart) of BM-MSCs into the skeletal muscle.

As previously described [13], the cells were isolated, expanded, and characterized in clinical-grade porcine MSCs. The cells were delivered (from passage 0 of the first bone marrow collection, BM1) locally into the wound bed. Each injection consisted of 0.5–1 mL containing 5–10 × 10^6^ MSCs, for a total treatment dose of 50–72 × 10^6^ BM-MSCs. The first injection was performed in the muscle before removal of the flap to cover the entire wound. A second local administration of 50–72 × 10^6^ MSCs (from passage 1 of BM1, volume 0.5–1 mL, 5–10 × 10^6^ MSCs) took place around the flap and in the muscle under it 1 week after surgery. Similarly, the third and fourth MSC doses (passage 0 and 1 from BM2) were injected 2 and 3 weeks after surgery, respectively (Fig. 1a). We previously report [13] that these cells were positive (> 90%) for CD90, CD29, CD44, and SLA-1 surface markers and cells differentiated into adipocytes, osteoblasts, and chondrocytes when cultured in medium that was simultaneously osteogenic, adipogenic, and chondrogenic.

A piece of skeletal muscle from animals in each treatment condition group (nonirradiated (C), irradiated (Irr), flap-only, and flap-MSC) was sampled close to the irradiated muscle at the time of surgery for the irradiated group and close to the muscle scar 1 year after surgery and treatment for the flap-only and flap-MSC groups. A piece of skeletal muscle from each animal was sampled before irradiation for the nonirradiated (C) group.

### Histological and immunohistochemical analysis

Freshly isolated muscle biopsies collected from the irradiated area at the time of surgery and from close to the scar 1 year after surgery and treatment were fixed in 4% paraformaldehyde and embedded in paraffin. Sections 5-μm-thick were dewaxed and hydrated; endogenous peroxidase was blocked with 3% hydrogen peroxide for 10 min, and nonspecific binding was blocked with a protein blocker (DakoCytomation, Trappe, France). H&E staining was performed for the overall morphological study, collagen deposition was detected by Sirius red staining, and Alizarin red staining was performed to identify areas of calcification, all according to standard methods. A pretreatment method using heat-induced epitope retrieval was used for the primary Col3a (Ab7778), slow myosin heavy chain (sMHC) (Ab11083), fast myosin heavy chain (fMHC) (Ab91506), and CD34 (Ab150060) antibodies; proteinase K (DakoCytomation) pretreatment was used for calprotectin S100A9 (Ab62227), Arg-1 (AV45672, Sigma), and Von Willebrand factor antibodies (A0082, Dako). The EnVision^+^ System (horseradish peroxidase, HRP) (DakoCytomation) was used as a secondary reagent for all immunostained sections. The color reaction was developed with the NovaRED™ kit (Vector Laboratories Inc., Burlingame, CA) and counterstained with Meyer’s hemalum. Staining for CD34 was developed with Histogreen substrate (E109; Abcys), and sections were counterstained with nuclear fast red (H-3403; Vector Laboratories). Arg-1 and Von Willebrand factor immunofluorescence staining were performed with goat Alexa 488 (Molecular Probes) and a goat Alexa 568 (Molecular Probes) for calprotectin S100A9 immunofluorescence. Cell nuclei were counterstained by Vectashield mounting medium with DAPI (Vector).

### Monocyte/macrophage polarization

Peripheral blood samples (10 mL in EDTA tubes) were collected before irradiation, the day of surgery (Irr group), and 2, 3, 4, 6, and 12 months after the flap surgery with and without BM-MSC treatment. Red blood cells were lysed with ACK lysis buffer (Life Technologies, France). Cells were washed three times and cultured in RPMI-1640 supplemented with 10% FBS and antibiotics (penicillin/streptomycin, Invitrogen) and 100 ng/mL of M-CSF (R&D Systems, France) at 37 °C in a humidified atmosphere containing 5% CO_2_ for 7 days to induce full macrophage differentiation and maturation. Macrophage polarization was obtained by removing the culture medium and culturing cells for an additional 24 h in RPMI-1640 supplemented with 10% FBS and antibiotics (penicillin/streptomycin, Invitrogen) and 100 ng/mL LPS (derived from *Escherichia coli* 0111:B4, Invivogen) plus 20 ng/mL IFN-γ (for M1 polarization, M(LPS-IFN-γ)) or 20 ng/mL IL-4 (for M2 polarization, M(IL-4)) (R&D Systems).

### Real-time PCR analysis

Total RNA was extracted from muscle with the RNeasy Mini kit (Qiagen), and cDNA was prepared with the SuperScript RT Reagent Kit (Applied Biosystems). Real-time PCR was performed on an ABI Prism 7000 Sequence Detection System. Syber chemistry (Life Technologies) was used to amplify PCR, with the specific primers listed in Table 1. All other Taqman primers and probes came from Life Technologies. Data were analyzed by the 2^−ΔΔCt^ method, with normalization to the Ct of the GAPDH (glyceraldehyde 3-phosphate dehydrogenase) housekeeping gene.

**Table 1:** Swine primers for real-time PCR

	Forward	Reverse
Col1a2	5′-CAGAACGGCCTCAGGTACCA-3′	5′-CAGATCACGTCATCGCACAAC-3′
Col3a1	5′CCTGGACTTCCTGGTATAGC-3′	5′-TCCTCCTTCACCTTTCTCAC-3′
TGF-β	5′-GCACGTGGAGCTATACAGA-3′	5′-ACAACTCCGGTGACATCAAA-3′
eNos	5′-GGCATCGCCAGAAAGAC-3′	5′-CATCACGGTGCCCATGAGT-3′
VEGF	5′-CCATGCAGATTATGCGGATCA-3′	5′-TCTCTCCTATGTGCTGGCCTTG-3′
iNOS	5′-CGTTATGCCACCAACAATGG-3′	5′-GAGCTGGAGCGTTCCCAGACC-3′
IL-10	5′-ACCAGATGGGCGACTTGTTG-3′	5′-TCTCTGCCTTCGGCATTACG-3′
GAPDH	5′-GACCCCTTCATTGACCTCCAC-3′	5′-TCCCATTCTCAGCCTTGACTG-3′

### Statistics

Data are expressed as means ± SEM. We used one-way analysis of variance (ANOVA) and then a Bonferroni post-test to determine the significance of differences. *P* values less than 0.05 were considered statistically significant.

## Results

### Effect of BM-MSCs on remodeling

H&E staining of transverse muscle sections showed a uniform size and polygonal shape with peripheral nuclei in the nonirradiated myofibers (Fig. 1c). In contrast, at 100 days after irradiation exposure, massive destruction was visible, with the loss of normal skeletal muscle architecture and the atrophy and angulation of variable-sized skeletal muscle fibers. The quantity of connective tissue was greater, and a large area of inflammatory cells had accumulated, replacing the damaged skeletal muscle fibers. One year post-surgery, the skeletal muscle architecture of the flap-only group had been restored, but there was a large area of fatty cell infiltration, which was absent in the flap-MSC group. Additional specific staining with Alizarin red showed calcification of myofibers on the day of surgery, which persisted 1 year post-surgery in the flap-only group (Fig. 1d). This calcification, which occurred predominantly in degenerated myofibers, was not observed in the flap-MSC animals.

**Fig. 2:**
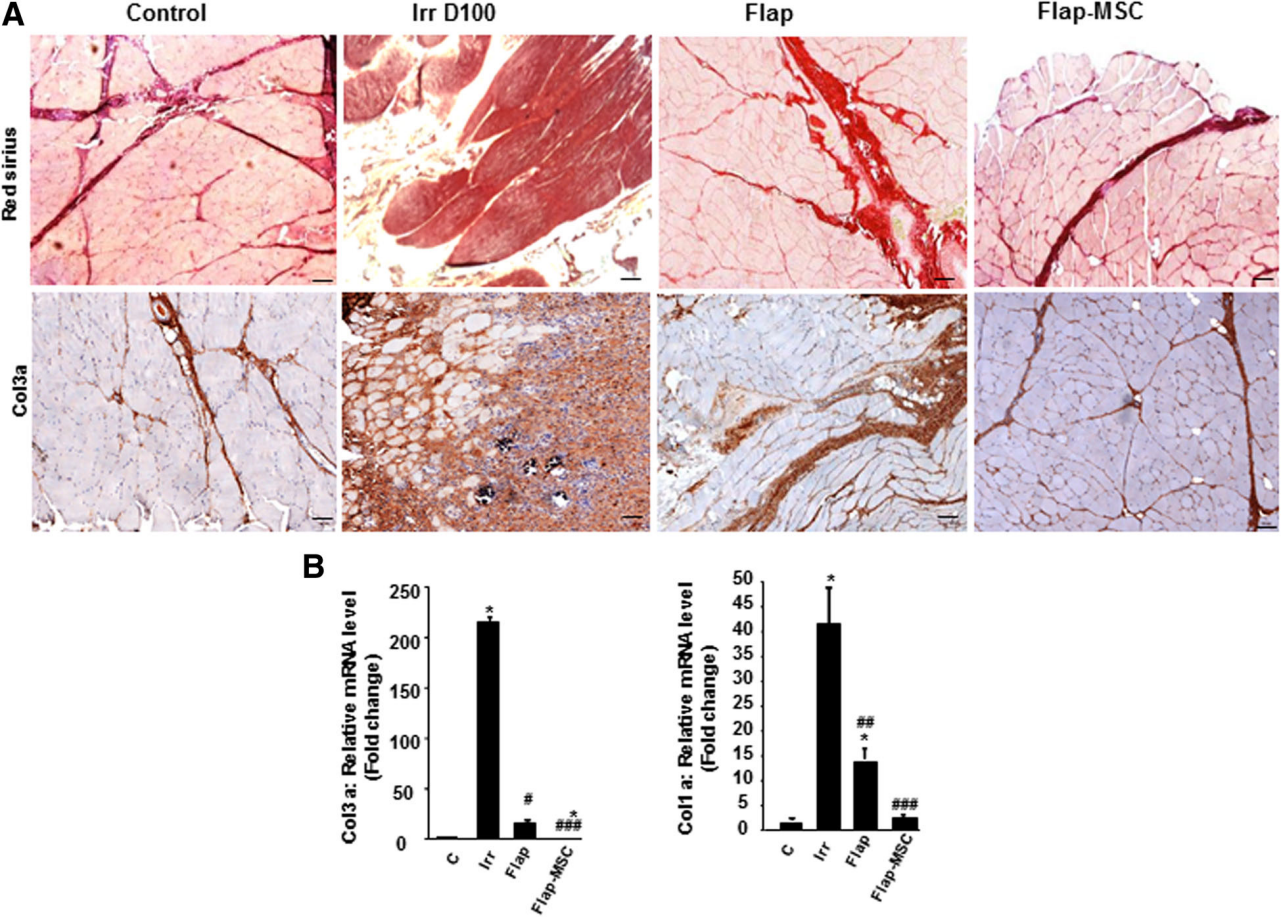
Effect of MSC treatment on deposition of extracellular matrix (ECM) components. **a** Representative whole-muscle cross-sections stained with Picro Sirius red to identify scar tissue (dark red) and Collagen-3 immunostaining of nonirradiated skeletal muscle (control group), irradiated skeletal muscle on the day of surgery (Irr D100 group), and 12 months after flap surgery with (flap-MSC group) and without (flap group) MSC treatment showed a net reduction of Col3 staining with the MSC treatment. Scale bar 100 μm. **b** Real-time-PCR of Col3a and Col1a. The flap-MSC group exhibited a net reduction of Col3a and Col1a expression compared with the irradiated and flap groups. Data are expressed relative to control skeletal muscle and normalized to GAPDH. Results are expressed as means ± SEM. *P* values were calculated by ANOVA with Bonferroni correction, *P < 0.001; compared with nonirradiated skeletal muscle; ^#^*P* < 0.05; ^##^*P* < 0.01; ^###^*P* < 0.001compared with irradiated-untreated controls

In severe injuries, defects or delays in the muscle regenerative process are frequently associated with aberrant remodeling of the ECM, which leads to an increase in fibrous tissue formation [8]. To evaluate this, skeletal muscle sections were stained with Sirius red, revealing a large area of collagen deposition in the muscle section on the day of surgery (day 100 post-irradiation) (Fig. 2a). In flap-MSC animals, the level of collagen deposition was similar to that in the nonirradiated muscle, while it remained elevated in the animals without MSC treatment. To confirm the effect of MSC treatment on the reduction of collagen deposition, we immunostained muscle sections with a specific antibody against ECM protein collagen 3a (Col3a). Figure 2b depicts a relative overabundance of Col3a on the day of surgery. At 1 year after surgery, the Col3a staining was still marked in the flap-only group, while in the flap-MSC group, it was similar to that in the nonirradiated muscle. Real-time PCR confirmed both overexpression of Col3a (215-fold, *P* < 0.001) and Col1a (42-fold, *P* < 0.001) in irradiated muscle on the day of the surgery, compared with nonirradiated muscle (Fig. 2c). Although reduced, expression of Col3a and Col1a remained significantly elevated (by factors of 14 and 15, respectively; *P* < 0.001) in the flap-only group at 1 year after surgery, compared with nonirradiated muscle. Only MSC treatment associated with flap surgery normalized their expression.

### Effect of BM-MSCs on the regeneration of different types of myofibers

**Fig. 3:**
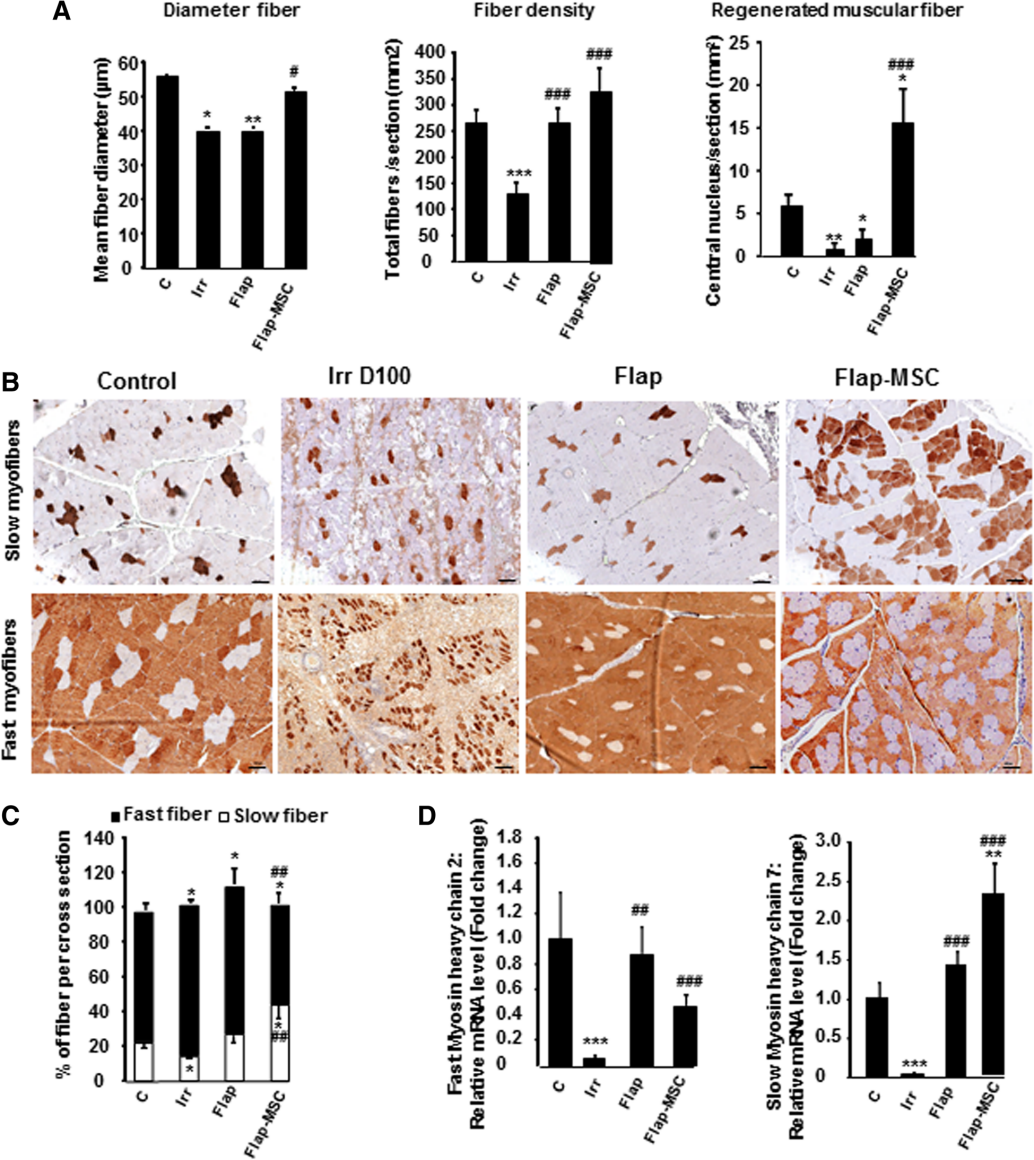
Effect of MSCs on myofiber regeneration. **a** Quantification of muscle fiber diameter, densities (calculated by normalizing the total number of fibers to section area), and number of regenerated fibers (identified by their central nuclei). **b** Immunostaining of the slow- and fast-twitch myofibers. **c** Percentage of each fiber type in nonirradiated skeletal muscle (control group), irradiated skeletal muscle on the day of surgery (Irr D100 group), and 12 months after flap surgery with (flap-MSC group) and without (flap group) MSC treatment. Scale bar 100 μm. **d** Real-time-PCR of fast myosin heavy chain 2 and slow myosin heavy chain 7. Data are expressed relative to control skeletal muscle and normalized to GAPDH. Results are expressed as means ± SEM. *P* values were calculated by ANOVA with Bonferroni correction, *P < 0.05; **P < 0.01; ***P < 0.001 compared with nonirradiated skeletal muscle; ^#^*P* < 0.05; ^##^*P* < 0.01; ^###^*P* < 0.001 compared with irradiated-untreated controls

To determine if MSC treatment and flap surgery restored muscle structure, myofiber diameter and density and the number of regenerated myofibers were quantified in histological cross-sections (Fig. 3a). At D100 (day of surgery), irradiated, compared with nonirradiated, muscle showed significantly lower myofiber diameters (27%, *P* < 0.05) and density (52%, *P* < 0.001). At 1 year, surgery alone did not restore myofiber diameter (a decrease of 29% in the flap-only group), but the MSC treatment with surgery did result in the restoration of myofiber diameter similar to that in nonirradiated muscle. Analysis of myofiber density showed a normalization in both flap-surgery groups. The number of regenerated myofibers was determined by counting their centrally located nuclei within the muscle cross-sections and was significantly lower (decrease of 87%, *P* < 0.001) in irradiated compared with nonirradiated muscle. The flap surgery did not modify this decrease significantly, but the flap-MSC group had a number of regenerated myofibers per section area 2.6 times higher (*P* < 0.05) than the nonirradiated muscle. These results suggest that the MSC injections enabled the maintenance of a high rate of regenerated myofibers and thus contributed to the increase of myofiber diameter and density.

Skeletal muscle myofibers come in three categories: fast-twitch, slow-twitch, and mixed (expressing both fMHC and sMHC) [14]. We investigated whether MSC treatment modified the muscle fiber phenotype. In Fig. 3b, fiber quantification showed that 22 ± 3% of the myofibers in nonirradiated muscle were positive for sMHC (Fig. 3c). On the day of surgery, the slow-twitch myofibers appeared to have been more vulnerable than the fast-twitch fibers, with only 14% of persistent slow myofibers for 86% of fast ones in irradiated muscle. One year after surgery, the level of slow-twitch myofibers had increased, by 27% in the flap-only group and by 44% in the flap-MSC group. In particular, in the flap-only group, the total percentage of myofibers exceeded 100% of that in the nonirradiated group and revealed the presence of mixed-type myofibers expressing both fMHC and sMHC; these were not present in the flap-MSC group. In addition, MSC treatment produced a shift of fast-twitch to slow-twitch myofibers, which was confirmed by the demonstration with real-time PCR of an increase of sMHC-7 and a decrease of fMHC-2, compared with the control and irradiated groups (Fig. 3d). Together, these data show that the substantial atrophy induced by irradiation and characterized by the variation in both size and density of fibers was not significantly restored 1 year after flap surgery alone, while the MSC treatment enhanced fiber regeneration, contributing to the restoration of their size and density and associated with a higher prevalence of slow fibers.

### MSC injections accelerated the restoration of vascular structures

**Fig. 4:**
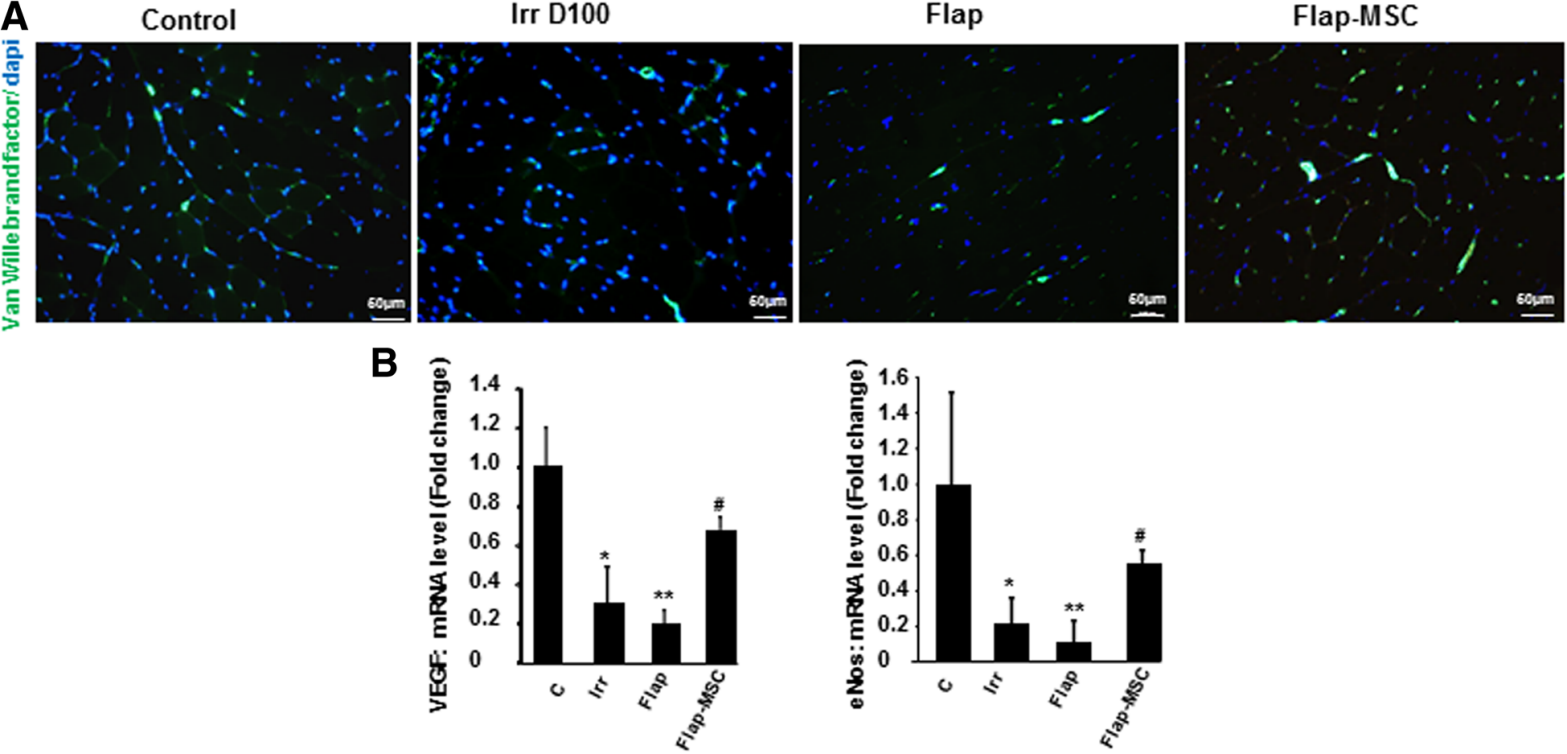
MSC injections accelerated vascular restoration after flap surgery. **a** Representative immunostaining of Van Willebrand factor. **b** Real-time expression of angiogenic factors VEGF and eNOS. Scale bar 50 μm. Results are expressed as means ± SEM. *P* values were calculated by ANOVA with Bonferroni correction, *P < 0.05; **P < 0.01 compared with nonirradiated controls; ^#^*P* < 0.05 compared with irradiated controls; ^#^*P* < 0.05 compared with irradiated-untreated controls

Revascularization is required for functional muscle recovery and markedly successful muscle regeneration. Figure 4 shows the immunostaining of Von Willebrand factor (vWF), an endothelial cell marker used to determine angiogenesis. On the day of surgery, irradiated muscles showed notably lower levels of positive staining for vWF than nonirradiated muscle (Fig. 4a). One year later, the number of vascular structures remained limited in the flap-only group, whereas it was completely restored in the flap-MSC group and similar to that of nonirradiated muscle. The loss of vessels on D100, which persisted for 1 year afterwards, was confirmed by repression of angiogenic factors such as VEGF and eNos, compared with nonirradiated muscle (Fig. 4b). In the flap-MSC muscles, vessel restoration was associated with the normalization of VEGF and eNos expression.

### Modulation of CD34^+^ cells by MSC treatment

**Fig. 5:**
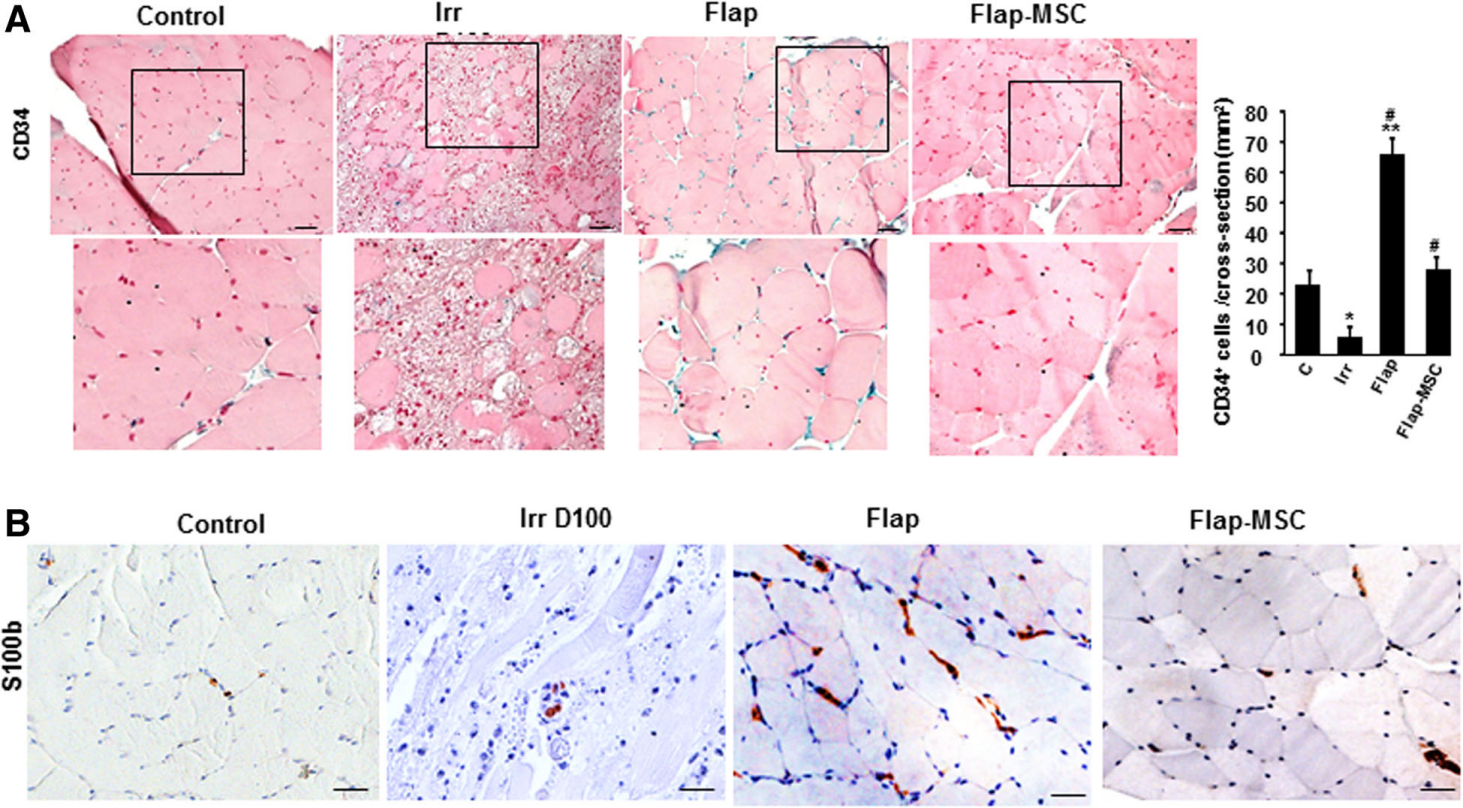
MSC controlled the repair process. **a** Modulation of CD34^+^ cells by MSC treatment. Representative CD34 immunostaining (better illustrated in the magnified view in the box) and real-time expression. **b** S100β representative immunostaining of nonirradiated skeletal muscle (control group), irradiated skeletal muscle on the day of surgery (Irr D100 group), and 12 months after flap surgery with (flap-MSC group) and without (flap group) MSC treatment. Scale bar 100 μm. Data are expressed relative to control skeletal muscle and normalized to GAPDH. Results are expressed as means ± SEM. *P* values were calculated by ANOVA with Bonferroni correction, *P < 0.05; **P < 0.001 compared with nonirradiated skeletal muscle; ^#^*P* < 0.001 compared with irradiated-untreated controls

CD34, which is expressed by both satellite and endothelial cells, is necessary for efficient muscle regeneration in response to both acute and chronic damage. Particularly needed for the early myogenic stages, CD34 disappears from the satellite cell surface before mature myofibers appear [15, 16]. Immunostaining showed a drastic loss of CD34^+^ cells in the irradiated, compared with nonirradiated, muscle on the day of the surgery (Fig. 5a). One year after flap surgery alone, the number of CD34^+^ cells had risen substantially, mainly at the periphery of myofibers, and was much higher than that of nonirradiated muscle. On the contrary, the number of these cells was normalized after flap-MSC treatment. The quantification from the cross-sections confirmed the significant enhancement of CD34^+^ cells in the flap-only group compared with nonirradiated (3-fold, *P* < 0.001) and their normalization following MSC treatment (Fig. 5b).

S100B had regulatory effects on both myoblast differentiation and the M1/M2 macrophage switch. Because rapid clearance of S100B after acute injury is required for timely resolution of inflammation and muscle regeneration [17], we used immunostaining to examine its expression in this animal model (Fig. 5c). The nonirradiated muscle showed low staining for cell-associated S100B, and the irradiated almost none at all on the day of surgery. A year later, the staining was highly marked in the muscle of the flap-only group. In the flap-MSC group, however, it was similar to nonirradiated muscle. Thus, MSC treatment probably contributed to the acceleration and stabilization of muscle regeneration.

### MSCs regulate the M1/M2 balance of infiltrating macrophages

**Fig. 6:**
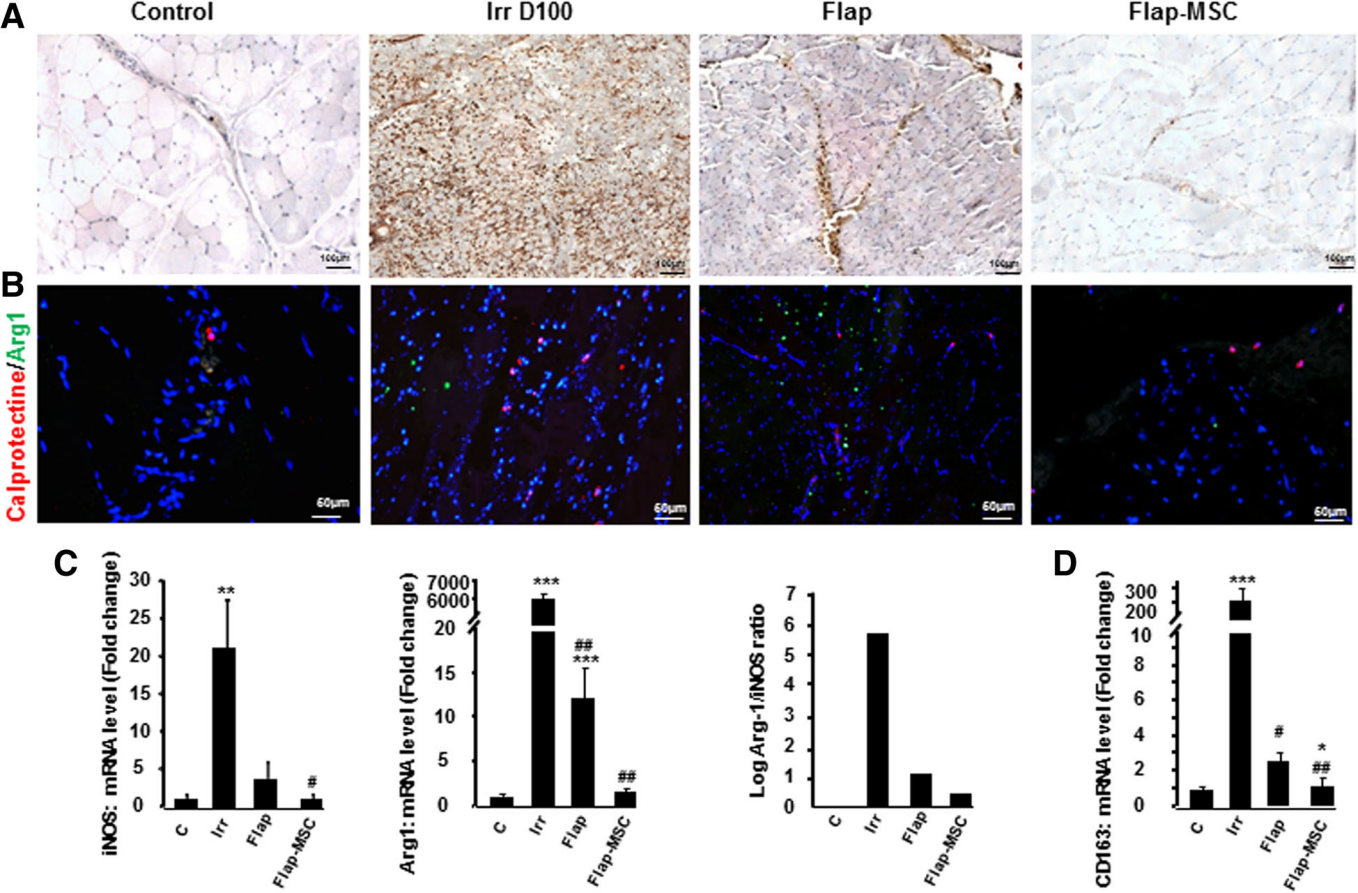
MSC treatment modulated macrophage infiltration and phenotype. **a** Representative immunostaining for calprotectin-positive macrophages. **b** Anti-calprotectin staining and anti-Arg-1 staining on muscle section of nonirradiated skeletal muscle (control group), irradiated skeletal muscle on the day of surgery (Irr D100 group), and 12 months after flap surgery with (flap-MSC group) and without (flap group) MSC treatment. **c** Real-time-PCR of iNOS, Arg1, and iNOS/Arg-1 mRNA level ratio as an index of M1/M2 activity balance. **d** Real-time-PCR of CD163 gene related to M2 polarization. Data are expressed relative to control skeletal muscle and normalized to GAPDH. Results are expressed as means ± SEM. *P* values were calculated by ANOVA with Bonferroni correction, *P < 0.05; **P < 0.01; ***P < 0.001 compared with nonirradiated skeletal muscle; ^#^*P* < 0.05; ^##^*P* < 0.001 compared with irradiated-untreated controls

Macrophages are crucial in muscle regeneration due to their involvement in muscle fiber maturation, resolution of inflammation at later stages of regeneration [18], and angiogenesis for the establishment of the capillary network necessary for this regeneration [19]. Calprotectin, which reflects macrophage cell infiltration, is detected with the MAC387 antibody; its use revealed a significant increase in the number of macrophages among the necrotic fibers on the day of surgery (Fig. 6a). Macrophage infiltration was considerably reduced 1 year after flap surgery but remained associated in particular with calcified deposits. In the flap-MSC group, macrophage infiltration was mostly present in the vessels and was similar to that in nonirradiated muscle.

Modification of the frequency and phenotype of macrophages affects wound healing as well as tissue fibrosis [20]. We used immunofluorescence staining of calprotectin to detect the presence of M1 proinflammatory macrophages and arginase-1 (Arg1) for M2 alternatively activated macrophages in muscle. On the day of surgery, this staining showed the coexistence of higher levels of both M1 and M2 macrophages than in nonirradiated muscle (Fig. 6b). A year later, both M1 and M2 macrophages remained present in the flap-only group, with a shift to M2 dominance. MSC treatment, however, both decreased the number of macrophages and balanced their M1/M2 phenotype. We also used real-time PCR to assess the expression of iNOS (a marker of the M1 proinflammatory macrophages) and Arg-1. As Fig. 6c shows, iNOS and Arg-1 were significantly overexpressed on the day of surgery compared with the nonirradiated group. A year after surgery, iNOS expression in both the flap-only and flap-MSC groups was drastically reduced. Arg-1 expression was normalized only with MSC treatment. Analysis of the ratio of Arg-1/iNOS mRNA levels, which serves as an indicator of the M2/M1 activity balance, showed this ratio skewed towards Arg-1 expression on the day of the surgery. Although the Arg-1/iNOS ratio decreased markedly in the flap-only group, only MSC treatment balanced it. Furthermore, analysis of CD163 expression (an M2 marker) confirmed the M2 shift on the day of surgery (200-fold; *P* < 0.001) and in the flap-only group (3-fold; *P* < 0.05), as well as its normalization in the flap-MSC group, compared with nonirradiated muscle (Fig. 6d).

**Fig. 7:**
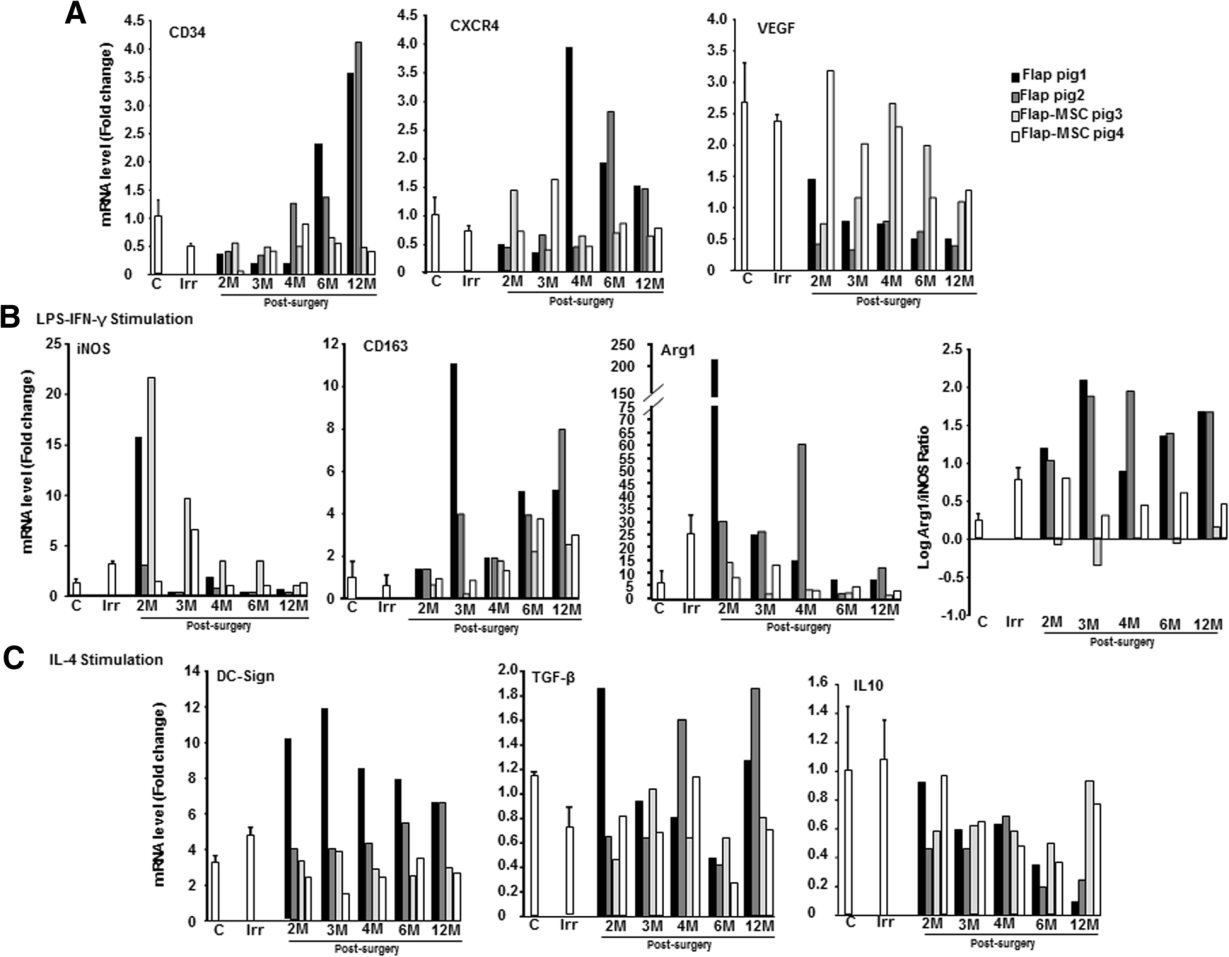
Course of gene expression from blood monocytes differentiated in different culture conditions from pigs before irradiation (C), on the day of surgery (Irr), and 2, 3, 4, 6, and 12 months after flap surgery with or without BM-MSC treatment. Macrophages were generated from blood monocytes in the presence of M-CSF. Real-time-PCR **a** in an unstimulated condition, **b** in an M1 condition after stimulation with LPS and IFN-γ, and **c** in an M2a condition, after stimulation with IL-4. Data are expressed relative to control skeletal muscle and normalized to GAPDH

As MSC treatment clearly affected the role of macrophages in wound healing, we sought to correlate the contribution of this treatment and the differentiated macrophage subtypes to the muscle kinetic repair process. It has previously been shown that the macrophages that accumulate in injured muscle are derived mainly from blood monocytes [21]. We therefore used M-CSF [22] to generate in vitro differentiated and matured macrophages from a blood monocyte population and followed their differentiation status during the wound healing. Using real-time PCR analyses, we showed first that CD34 and CXCR4 expression stabilized over time and were similar in the differentiated macrophages with M-CSF from the flap-MSC group and the nonirradiated group (Fig. 7a). In the flap-only group, however, both were overexpressed at 6 months. On the other hand, the drastic VEGF repression observed on the day of the surgery had improved at 3 months in the flap-MSC group, but not in the flap-only group. The activation state was assessed by gene expression linked to M(LPS-IFN-γ) macrophages, which reflects M1 activation: the ratio of Arg-1/iNOS mRNA levels (indicator of the M2/M1 activity balance) was similar over time in nonirradiated muscle and in the flap-MSC group; in the flap-only group, however, this ratio continued its M2 activity balance at 1 year, as on the day of surgery. In the M(IL-4) activation state, characterizing M2a activation, DC-SIGN, TGF-β, and IL-10 expression were normalized (at as early as 4 months for DC-SIGN) in the flap-MSC group, whereas the opposite effect was observed in the flap-only group: their expression increased. Taken together, these data show that the flap-only group maintained an elevated macrophage population in the muscle, with M2 dominant in the muscle tissue as well as in blood monocyte/macrophages, whereas the muscle macrophage population in the flap-MSC group was similar to that of the nonirradiated tissue, with an M1/M2 balance in both tissue and blood.

## Discussion

Medical management of severe musculocutaneous radiation syndrome requires surgical intervention for the debridement of necrotic tissue (skin and muscle). Even when skin excision is replaced by specific plastic surgery [13], the excision of fibrotic muscle areas, which implies the loss of a perhaps substantial volume of skeletal muscle, may be harmful. Validated in several animal models of muscle trauma, MSCs have emerged as promising treatment of enhancing muscle regeneration [11]. The aim of this study was therefore to assess the quality of long-term muscle regeneration after resection of irradiation-induced necrotic tissue, placement of a vascularized flap, and MSC injection.

The preclinical minipig model has showed that irradiation of musculocutaneous tissue induces major structural impairment of muscle fibers, characterized 3 months after exposure by myofiber atrophy, increased interstitial space between the formation of myofibers and fibrotic scar tissue, and disruption of the inherent vascular network. The risk of impaired wound healing is particularly important in irradiated tissue, where the unpredictable spatiotemporal course of inflammatory waves [23] increases the incidence of fibrosis, which is especially predominant after resection of fibrotic and necrotic areas [3]. If untreated, scar tissue can establish a permanent presence in the muscle tissue, potentially inhibiting the formation of new or the fusion of existing myofibers and thus leading to decreased muscle function. In this study, the proof of concept of repeated BM-MSC injections for the restoration of the muscle structure without the calcification and infiltration of fat cells that signal abnormal or impaired muscle regeneration [24] was brought using a model of irradiated minipig.

The extracellular matrix (ECM) played an important role during skeletal muscle regeneration and acted as a functional link between skeletal muscle and bone [6]. In particular, collagen 1 inhibits muscular regeneration and supports the production of more collagen [25]. Moreover Alexakis et al. demonstrated that type 1 collagen inhibits differentiation of myoblasts [25]. MSC treatment stabilized the production of both collagen 1 and 3. In addition, compared to the flap surgery alone, flap surgery with MSC treatment made possible the long-term maintenance of regenerated myofibers in the numbers and structures necessary to produce diameters and density similar to those of nonirradiated muscle. Without local MSC treatment, the myofibers remained smaller in diameter than with treatment; this suggests the delayed proliferation and/or the fusion of activated satellite cells.

The contractile characteristics of skeletal muscles are determined by the level and ratio of myosin heavy chain (MHC) subtypes. Principally, slow-twitch fibers (sMHC), which are fatigue-resistant and therefore ideal for low-intensity, long-lasting contractions, can be differentiated from fast-twitch fibers (fMHC), which show high velocity in shortening and low resistance to fatigue [14]. In this study, immunostaining and gene expression showed a difference between the levels of slow- and fast-twitch myofibers, depending on the MSC treatment. MSCs favored the generation of slow-twitch myofibers, while the flap-only group had a higher prevalence of fast-twitch, as well as hybrid-type, myofibers. MSC treatment appeared to enhance in priority the conversion of fast-twitch to slow-twitch fibers during skeletal muscle repair. This conversion involved morphologic and biochemical changes that modified contractile properties and endurance capacity. In various atrophic conditions, the fast-twitch myofibers appear more vulnerable than the slow-twitch ones and thus provide evidence of the protective effects of the slow gene program [26, 27]. Thus, different factors (e.g., microenvironment, heterogeneous satellite cells) might affect the emergence of fiber types in regenerating muscles [28]. Accordingly, we cannot rule out the possibility that MSCs can act indirectly on ECM: by specifically targeting satellite cells, they may give rise to myotubes containing substantial amounts of slow-twitch myofibers, depending on whether the satellite cells come from a type 1 (sMHC) fiber [29].

Satellite cells are the primary source of new myogenic nuclei for skeletal muscle regeneration. These cells first exit their normal quiescent state to start proliferation; after several rounds of proliferation, most of them have differentiated and fused to form new myofibers or repair damaged ones. Alfaro et al. [15] showed that when satellite cells lack CD34, they encounter significant delay in their progression through the myogenic program. Our results show that, although the flap-only group had a reduction in both fiber size and frequency of central nuclei, which are signs of regenerative myofibers, they also had a higher number of CD34^+^ cells, in contrast to the flap-MSC group, in which this number was normalized, i.e., reduced. It has previously been reported that CD34 is required for myogenic progenitor cells and satellite cells to induce the early stages of myogenic progression. CD34 acts by mediating the activation, proliferation, and motility of myogenic cells [15] but disappears from the satellite cell surface before the mature myofibers emerge. The CD34^−^ cells appear to represent a “primed” satellite cell population available for injury response and that can upregulate CD34 or revert back to establish a reserve state, according to the muscle microenvironment [16]. As the presence or absence of CD34 is reversible, depending on injury status, our data suggest that a year after surgery without MSC treatment, muscle regeneration was still at the first step of myogenic activation, demonstrated by the massive number of CD34 cells present. The disappearance of these cells with MSC treatment may be a sign that regeneration has been accomplished. This point is reinforced by the fact that successful muscle healing required re-establishment of the vascular network. Analysis of muscle section staining with Van Willebrand factor and angiogenesis-related genes (e.g., VEGF, eNos) confirmed the long-lasting stabilization of the vascular network in the flap-MSC group, although it was not yet restored at 1 year after surgery in the flap-only group.

Another marker of regeneration was the level of S100β, a calcium-binding protein released by damaged myofibers and infiltrated macrophages; it is required for correct timing of muscle regeneration. Its persistence at high levels, however, weakens regeneration [17] and causes the transition of myoblasts into adipocytes [30]. Also at a year after surgery, the flap-only group showed marked S100β immunostaining and fatty tissue in the muscle, whereas the level of this immunostaining in the flap-MSC group was similar to that in nonirradiated muscle and, like this control, showed no fat deposition. Excess S100β expression in activated satellite cells is detrimental for muscle regeneration: its mitogenic effect interferes with the reconstitution of the satellite cell reserve pool, without which recurrent muscle regeneration leads rapidly to the depletion of the pool and finally delays myoblast proliferation and differentiation [1]. In addition, high extracellular S100β levels promote fibrotic tissue deposition and thereby jeopardize regeneration [17]. We can thus hypothesize that MSC treatment regulated S100β levels to prevent excessive expansion of activated satellite cells, which would lead to defective reconstitution of both damaged tissue and the quiescent satellite cell pool.

Macrophages are essential for muscle repair, through their delivery of trophic factors to growing skeletal muscle precursors and young fibers. It has previously been reported that M1 macrophages, which create a proinflammatory environment, increase myoblast proliferation, decrease fibroblast collagen production [31], but delay myoblast differentiation. It is the spontaneous shift from M1 towards M2a anti-inflammatory macrophages, influenced by the microenvironment, that promotes myoblast differentiation [32]. Macrophages in injured muscle do not exhibit either a strictly M1 or M2a phenotype but rather a mixture of both M1- and M2a-associated markers; most markers subsequently decrease. When we specifically analyzed macrophage polarization in this study, using both immunostaining and expression of a specific M1 marker (iNOS) and M2 marker (Arg1) and their ratio, we found that the flap group maintained a high density of macrophages and shifted towards a M2 prevalence, compared with the irradiated group. Inversely, the number of macrophages in the flap-MSC group was restricted, and the M1/M2 phenotype was balanced. Indeed, when the differentiation and fusion of fibers are completed [21], the number of macrophages drops to a very low level, which suggests that muscle regeneration was nearly completed in the flap-MSC group. In the flap-only group, on the other hand, the proliferation and differentiation steps of regeneration could take place without MSC treatment because of the large M2 predominance. A previous report showed that M2 macrophages increase the production of collagen by fibroblasts, which disrupts the formation of new fibers [31]. In our study, the permanent deposition of collagen 1 and 3, the decrease in myofiber diameter, the failure to regenerate muscle fibers, the reduced number of capillaries, and the fat accumulation 1 year post-surgery suggest that, in the absence of MSC treatment, the prevalence of M2 macrophages might play a role in abnormal regeneration.

Wound macrophages are derived mainly from circulating monocytes, which acquire an activated phenotype with changes in the associated markers on arrival at the injury site [21]. First, we showed that under ex vivo non-stimulated conditions, only MSC treatment contributed to the macrophages’ overexpression of VEGF, observed from 3 months post-surgery; this observation may be predictive of the stabilization of the vascular network at 1 year post-surgery. We showed that, under M1 stimulation by LPS/IFN-γ, macrophages isolated from the flap-MSC group acquired a proinflammatory phenotype more quickly (from 3 months post-surgery) than those in the flap-only group. Inversely under M2a conditions, resulting from stimulation with IL-4, the flap-MSC group down-repressed TGF-β and lose their anti-inflammatory spectrum faster. Additionally, Arg1, considered as an M2a marker, may be expressed in the M1 as well as the M2a phenotype, as confirmed by wound macrophages expressing both high and low levels of M1-spectrum markers [33]. These ex vivo macrophage differentiation results agree with our in vivo findings of long-term M2 predominance in the flap group, without MSC treatment, compared with strong limitation of macrophage infiltration and a switch from the M2 spectrum towards an M1/M2 balance in the flap-MSC group. Interestingly, in our study during the M2 stimulation, MSC treatment normalized expression of IL-10, which is a powerful anti-inflammatory cytokine that can suppress both M1 and M2a activation [34]. The importance of the macrophage phenotype shift in the regeneration of skeletal muscle that we report here strengthens the previous proof-of-concept study of in vitro-activated macrophages, where exogenous M1 macrophages decreased fibrosis and enhanced regeneration in injured muscle [35]. A previous report shows that a substantial portion of MSCs’ contribution to the regeneration of skeletal muscle is through mechanisms other than myogenic differentiation [36]. This demonstrates the attractiveness of their paracrine role as an explanation of their effect on tissue regeneration. Although such adult stem cells are involved in the regeneration of host skeletal muscle tissue, their functional contributions to muscle regeneration have not yet been clearly demonstrated.

On the same irradiated pigs, at the skin level, we have showed that BM-MSC injections were indispensable to the preservation of skin wound-healing quality after flap surgery for the radiation exposure [13]. BM-MSC treatment contributed to a skin mature ECM devoid of inflammation and enabling vascular stability, resulting in tissue remodeling with less scar formation and long-term maintenance of skin wound healing. But irradiation exposure involved damage at both muscle and skin. Skeletal muscle holds significant regenerative potential but is incapable of large restoring tissue loss caused by severe injury. Skeletal muscle wound healing is dependent on complex interactions between fibroblasts, myofibroblasts, and myogenic cells, and the healthy skin is a cellular scaffold with surrounding ECM, and trophic factors promote a constructive, functional skeletal muscle response for the injury skeletal muscle. A recently established transdifferentiation of dermal fibroblasts into skeletal muscle [37] reinforces the close relation between optimal skeletal muscle regeneration and the requirement of stable cutaneous coverage over time.

## Conclusion

In this study, the group treated by BM-MSCs (and flap surgery) showed clearly established muscle regeneration at 1 year after treatment. BM-MSC therapy may act on several levels, including by generating growth factors that stimulate VEGF, which is involved in angiogenesis and promotes both muscle cell regeneration and growth, and by acting on the satellite cell pool and accelerating the macrophage shift that promotes muscle regeneration. Thus, cell therapy by local BM-MSC injection is a safe and effective way to improve long-term recovery of irradiation-induced skeletal muscle damage without signs of degeneration.

## Abbreviations

Arg1: Arginase 1
BM: Bone marrow
BM-MSC: Bone marrow-derived mesenchymal stromal cell
ECM: Extracellular matrix
eNOS: Endothelial nitric oxide synthases
iNOS: Inductible nitric oxide synthases
MSC: Mesenchymal stromal cell
sMHC: Slow myosin heavy chain
fMHC: Fast myosin heavy chain
RT-PCR: Real-time quantitative PCR
VEGF: Vascular endothelial growth factor
